# The Expanding Therapeutic Potential of Deucravacitinib Beyond Psoriasis: A Narrative Review

**DOI:** 10.3390/jcm14051745

**Published:** 2025-03-05

**Authors:** Chul-Hwan Bang, Chul-Jong Park, Yoon-Seob Kim

**Affiliations:** 1 Department of Dermatology, Seoul St. Mary’s Hospital, College of Medicine, The Catholic University of Korea, Seoul 06591, Republic of Korea; 2 Department of Dermatology, Bucheon St. Mary’s Hospital, College of Medicine, The Catholic University of Korea, Seoul 14647, Republic of Korea

**Keywords:** deucravacitinib, TYK2 inhibitor, psoriasis, psoriatic arthritis, systemic lupus erythematosus, inflammatory bowel disease, palmoplantar pustulosis, autoimmune diseases, immune-mediated diseases

## Abstract

Deucravacitinib is an allosteric, selective tyrosine kinase 2 (TYK2) inhibitor that has demonstrated significant efficacy in the treatment of psoriasis. TYK2, a member of the Janus kinase (JAK) family, plays a critical role in intracellular signaling pathways for pro-inflammatory cytokines. Unlike traditional JAK inhibitors, which target active domains, deucravacitinib selectively binds to the pseudokinase domain of TYK2. This binding induces a conformational change that locks the enzyme in an inactive state, ensuring superior selectivity for TYK2 over JAK 1/2/3. This unique mechanism specifically inhibits key pro-inflammatory cytokines, including IL-12, IL-23, and type I interferons, critical in the pathogenesis of psoriasis and other immune-mediated diseases. As a result, deucravacitinib represents a promising option for targeted therapy in immune-mediated diseases and may reduce adverse events commonly associated with broader immunosuppressive treatments. Furthermore, its oral administration offers a convenient alternative to injectable biologics, potentially improving patient adherence and treatment satisfaction. This review highlights recent studies suggesting that deucravacitinib may also have therapeutic benefits in psoriatic arthritis, palmoplantar pustulosis, systemic lupus erythematosus, Sjogren’s disease, and inflammatory bowel disease. Given its expanding therapeutic potential, deucravacitinib may provide a safer and more effective alternative to current therapies, offering a tailored approach to treatment.

## 1. Introduction

The development of selective tyrosine kinase 2 (TYK2) inhibitors marks a significant advancement in the treatment of immune-mediated diseases, particularly psoriasis. TYK2, a member of the Janus kinase (JAK) family, plays a crucial role in immune and hematopoietic cell functions, including cell growth, survival, differentiation, and immune response regulation [1]. Unlike JAK1, JAK2, and JAK3, TYK2 uniquely mediates intracellular signaling for key pro-inflammatory cytokines such as interleukin (IL)-12, IL-23, and type I interferons [2], which are central to the pathogenesis of psoriasis and other immune-mediated disorders.

TYK2 forms signaling complexes with JAK1 and JAK2 but not JAK3. For example, type I interferons signal through a JAK1-TYK2 combination, while IL-12 and IL-23 utilize JAK2-TYK2 pairings for signal transduction [3]. Activation of these JAKs triggers receptor phosphorylation, creating docking sites for STAT (signal transducer and activator of transcription) proteins, which translocate to the nucleus to regulate immune activation and inflammation [4]. This positions TYK2 as a key regulator of both the IFN/Th1 and IL-23/Th17/IL-17 signaling pathways, which are critical in psoriasis and other immune-mediated diseases [4,5,6]. The IFN/Th1 pathway involves the differentiation of naïve T cells into Th1 cells, which produce IFN-γ, activating macrophages and enhancing antigen presentation [7]. The IL-23/Th17/IL-17 pathway involves the differentiation of naïve T cells into Th17 cells driven by IL-23. These Th17 cells produce IL-17, recruiting neutrophils and promoting inflammation [8]. Both pathways are implicated in autoimmune and inflammatory diseases.

Beyond its role in psoriasis, TYK2 mediates signaling for a broader range of cytokines involved in innate and adaptive immune responses, such as IL-6, IL-10, and IL-27, underscoring its potential relevance in various immune-mediated diseases [9,10,11]. Despite its established importance, the full spectrum of TYK2’s biological functions remains incompletely understood, warranting further investigation into its roles in immune regulation and disease pathogenesis.

Deucravacitinib, the first oral selective allosteric TYK2 inhibitor approved for psoriasis treatment, also shows potential for addressing other immune-mediated diseases. Emerging evidence suggests its utility in conditions such as psoriatic arthritis (PsA), palmoplantar pustulosis (PPP), systemic lupus erythematosus (SLE), Sjögren’s disease (SjD), and inflammatory bowel disease (IBD), underscoring its versatility in modulating immune pathways across multiple specialties.

This review summarizes the current evidence on deucravacitinib and explores its broader therapeutic potential, particularly in PsA, PPP, SLE, SjD, and IBD, by examining recent pivotal clinical data.

## 2. Mechanism of Action of Deucravacitinib

TYK2-mediated signaling plays a central role in both innate and adaptive immunity. Studies in human and mouse models have highlighted the critical functions of TYK2. For example, Tyk2-deficient mice, in which the Tyk2 gene has been disrupted, exhibit impaired growth inhibition of lymphocytes in response to interferon-alpha (IFN-α) and show limited IL-12-induced cellular responses of T cell function [12]. These findings underscore the importance of TYK2 in immune and inflammatory processes, particularly in regulating the IFN/Th1 and IL-23/Th17/IL-17 signaling axes. This role makes TYK2 inhibition therapeutically relevant for conditions like psoriasis and other immune-mediated diseases driven by IL-12 and IL-23.

Deucravacitinib represents a significant advancement in the field of targeted therapies for immune-mediated diseases. Unlike conventional Janus kinase (JAK) inhibitors, which target the highly conserved active kinase domains shared by all JAK family members, deucravacitinib uniquely binds to the regulatory (pseudokinase) domain of TYK2. This binding induces a conformational change, locking TYK2 in an inactive state and preventing downstream signaling [10]. This allosteric inhibition ensures exceptional specificity, achieving 100-fold to 2000-fold selectivity for TYK2 over JAK1, JAK2, and JAK3 in vitro [13].

Clinical trials have consistently demonstrated the efficacy of deucravacitinib in psoriasis, with patients showing substantial improvements in skin clearance and quality of life. Importantly, deucravacitinib has a favorable safety profile, with no significant associations with laboratory abnormalities such as cytopenias or elevated liver enzymes, nor does it increase the risk of opportunistic infections. Most adverse events (AEs) in clinical trials have been mild to moderate, further supporting its safety [14,15,16,17].

Deucravacitinib effectively addresses several limitations of traditional psoriasis therapies, such as cyclosporine and TNF inhibitors, which are often associated with systemic immune suppression, an increased risk of opportunistic infections, injection-related challenges, and inconsistent patient adherence. Its oral formulation, coupled with a relatively lower cost compared to biologics, enhances convenience and adherence, offering a practical and patient-friendly alternative to injectable biologics.

Deucravacitinib is approved for the treatment of adults with moderate-to-severe plaque psoriasis who are candidates for systemic therapy or phototherapy. It received approval from the Food and Drug Administration (FDA) in 2022, from the European Medicines Agency (EMA) in 2023, and from Korea’s Ministry of Food and Drug Safety (MFDS) in 2023. The approvals in these countries were based on the results of the phase 3 POETYK PSO-1 (NCT03624127) and POETYK PSO-2 (NCT03611751) clinical trials, which demonstrated its efficacy in treating moderate-to-severe plaque psoriasis. In Japan, deucravacitinib was approved in 2023 by the Pharmaceuticals and Medical Devices Agency (PMDA) for the treatment of adults with moderate-to-severe plaque psoriasis, generalized pustular psoriasis, and erythrodermic psoriasis, based on the phase 3 POETYK PSO-4 clinical trial (NCT03924427), which was conducted exclusively with Japanese patients.

While deucravacitinib is currently approved for psoriasis, emerging evidence suggests its potential in other immune-mediated diseases. Its unique mechanism of action and broad cytokine modulation position it as a promising therapeutic option for addressing unmet clinical needs across various specialties. Although the mechanism of action is well-characterized as a selective allosteric TYK2 inhibitor, further research is needed to fully understand its molecular interactions and downstream effects in diverse immune-mediated diseases. Continued studies will help optimize its therapeutic potential and identify additional indications for its use.

## 3. Deucravacitinib vs. JAK Inhibitors or Biologics

Deucravacitinib selectively targets the pseudokinase domain of TYK2, inducing a conformational change that locks the enzyme in an inactive state. This unique mechanism ensures superior specificity for TYK2 over JAK 1/2/3, differentiating it from traditional JAK inhibitors that target active kinase domains and may affect multiple JAK isoforms [10]. By selectively inhibiting TYK2, deucravacitinib modulates key pro-inflammatory cytokines, including interleukin IL-12, IL-23, and type I interferons, without disrupting broader immune functions mediated by other JAK family members [18]. This selectivity minimizes off-target effects commonly seen with broader JAK inhibition, such as immunosuppression and hematological toxicities, potentially leading to a better safety profile. Furthermore, JAK inhibitors and deucravacitinib may have distinct indications in current and future practices. For example, upadacitinib is approved for broader indications, including PsA, IBD, rheumatoid arthritis, ankylosing spondylitis, and axial spondyloarthritis, but not for psoriasis. Taken together, the differences in mechanism of action, selectivity, safety profile, and approved indications highlight the potential for deucravacitinib to have a distinct role in immunological diseases compared to JAK inhibitors.

In comparison to biologics for psoriasis, such as TNF-α inhibitors (e.g., infliximab, adalimumab), IL-12/23 inhibitors (e.g., ustekinumab), IL-17 inhibitors (e.g., secukinumab, ixekizumab), and IL-23 inhibitors (e.g., guselkumab, risankizumab) that target specific cytokines involved in inflammatory pathways, deucravacitinib offers a unique mechanism of action. A network meta-analysis has demonstrated that the efficacy of deucravacitinib in treating psoriasis is comparable to TNF-α inhibitors and IL-12/23 inhibitors, but lower than the more recent biologics of IL-17 inhibitors and IL-23 inhibitors [19]. While biologics have well-established safety profiles, they are associated with injection site reactions and rare systemic infections. Deucravacitinib’s oral administration may reduce these risks and improve patient adherence. Furthermore, deucravacitinib’s ability to modulate multiple cytokines provides a distinct advantage over biologics, which typically target a single cytokine, such as IL-17 or IL-23 inhibitors. This broader cytokine modulation may improve its therapeutic potential in complex immune-mediated diseases, where multiple inflammatory pathways contribute to pathogenesis.

## 4. Psoriasis

The pivotal phase 3 POETYK PSO-1 (n = 666) and PSO-2 (n = 1020) trials demonstrated robust efficacy of deucravacitinib in achieving Psoriasis Area and Severity Index (PASI) 75 and Static Physician’s Global Assessment (sPGA) 0/1 responses in patients with moderate-to-severe psoriasis (PASI ≥ 12, sPGA ≥ 3, body surface area (BSA) ≥ 10%) [7,8]. At week 16, the deucravacitinib 6 mg once-daily group showed significantly higher PASI 75 and sPGA 0/1 response rates compared to the apremilast 30 mg twice-daily group and placebo [14,15]. Long-term data from the POETYK PSO-LTE trial, the 3-year open-label extension of POETYK PSO-1 and PSO-2 trials (NCT04036435) demonstrated sustained efficacy, with 73.2% of patients achieving PASI 75 and 48.1% achieving PASI 90 by week 148, highlighting the durability of its therapeutic benefits [16].

Pooled safety analyses from the 52-week POETYK PSO-1 and PSO-2 trials revealed a favorable safety profile for deucravacitinib. Exposure-adjusted incidence rates (EAIRs) for AEs were comparable across treatment groups, with low rates of serious AEs and treatment discontinuations. Major adverse cardiovascular events and malignancies were not increased compared to placebo or apremilast, and laboratory parameters remained stable within acceptable ranges throughout the study [20]. In the POETYK PSO-LTE trial, the safety profile remained consistent, with a decline in EAIRs for AEs over time, indicating improved tolerability with prolonged use. Nasopharyngitis and upper respiratory tract infections were the most commonly reported AEs, while serious AEs and treatment discontinuations remained infrequent and stable [16].

Although not yet published, new 5-year results from the POETYK PSO-LTE trial showed that deucravacitinib maintains consistent safety and durable response rates in moderate-to-severe plaque psoriasis, with 46.3% of patients achieving PASI 90 at year 5, and no new safety signals observed [21].

## 5. Psoriatic Arthritis (PsA)

PsA is a chronic inflammatory arthritis that affects up to 30% of psoriasis patients and is characterized by joint inflammation, enthesitis, and dactylitis. Without timely intervention, PsA can lead to joint damage, disability, and significant reductions in quality of life [22]. Plaque psoriasis and PsA share a common T-cell-mediated immunopathologic mechanism involving activation of the IL-23/Th17/IL-17 axis [23]. Similarly to psoriasis, the pathogenesis of PsA involves a complex interplay of genetic, immunologic, and environmental factors, with key roles attributed to cytokines such as IL-12, IL-23, and IL-17 [24]. Although several therapies are available, many PsA patients remain inadequately treated due to safety concerns, inconvenient dosing regimens, or insufficient efficacy [25].

As a selective TYK2 inhibitor, deucravacitinib modulates the signaling pathways of IL-12 and IL-23, offering a promising therapeutic option for PsA [26]. In the IM011-084 trial, a phase 2 randomized controlled trial (NCT03881059), involving 203 patients with active PsA, deucravacitinib demonstrated significant efficacy and a favorable safety profile. Patients received placebo, deucravacitinib 6 mg once daily, or deucravacitinib 12 mg once daily. At week 16, American College of Rheumatology 20 (ACR-20) response rates were significantly higher in both deucravacitinib groups (52.9%; *p* = 0.0134 and 62.7%; *p* = 0.0004 for 6 mg and 12 mg, respectively) compared to placebo (31.8%) [27]. A significantly higher proportion of patients treated with deucravacitinib also achieved minimal disease activity (22.9%; *p* < 0.05 and 23.9%; *p* < 0.01 for 6 mg and 12 mg, respectively) compared to placebo (7.6%) [28]. Furthermore, treatment significantly reduced levels of biomarkers involved in PsA pathogenesis, including IL-17A, beta-defensin 2 (BD-2), IL-19, CXCL9, CXCL10, CRP, MMP-3, and collagen type 4 degradation markers (*p* < 0.01) [29].

Two 52-week phase 3 trials, POETYK PsA-1 (NCT04908202) and POETYK PsA-2 (NCT04908189), have evaluated the efficacy and safety of deucravacitinib in PsA, with preliminary results now publicly available. These trials enrolled over 1400 adults with active PsA and achieved their primary endpoint, showing a significantly higher proportion of patients achieving an ACR20 response at week 16 compared to placebo. The safety profile was consistent with previous studies. These findings suggest that deucravacitinib may become the first oral TYK2 inhibitor approved for PsA. Detailed results are expected to be published soon [30].

Future research should prioritize direct comparisons of deucravacitinib with existing therapies, investigation of combination regimens, and identification of patient subgroups most likely to benefit. Such efforts are crucial for optimizing treatment strategies and improving clinical outcomes.

## 6. Palmoplantar Pustulosis (PPP)

PPP is a chronic, relapsing, inflammatory skin disease characterized by the presence of multiple sterile pustules that develop from vesicles on erythematous, scaling skin and are confined to the palms and soles [31]. While PPP and psoriasis vulgaris share some inflammatory pathways, such as the involvement of the IL-23/IL-17 axis, PPP is not exclusively driven by this axis, as is the case with psoriasis vulgaris. Instead, it is strongly associated with various triggers, including smoking and infectious agents [32].

The IL-23/IL-17 pathway plays a critical role in sustaining chronic inflammation in PPP, making IL-23 inhibitors promising therapeutic targets [33]. Recently, IL-23 inhibitors such as guselkumab and risankizumab have been approved for adult patients with moderate-to-severe PPP [34,35]. Additionally, a small case series reported that upadacitinib, a selective JAK1 inhibitor, significantly improved PPP severity, suggesting its potential as a promising agent for this condition [36,37]. These findings indicate that deucravacitinib, a selective TYK2 inhibitor, may also be an effective treatment option for PPP.

A retrospective analysis of five patients with refractory PPP treated with deucravacitinib (6 mg/day) revealed initial worsening in most patients; however, three experienced improvement by week 16. Although AEs, primarily viral and bacterial infections, were reported, the study highlights deucravacitinib’s potential as a treatment for PPP. Nevertheless, larger trials are needed to confirm its efficacy and safety [38].

## 7. Systemic Lupus Erythematosus (SLE)

SLE is a chronic autoimmune disease characterized by widespread inflammation and tissue damage that affects multiple organ systems, including the skin, kidneys, joints, and central nervous system [39]. Its pathogenesis involves a combination of genetic predisposition, environmental triggers, and dysregulated immune responses, with hallmark features such as the production of autoantibodies (e.g., ANA) and immune complex formation contributing to tissue injury [40]. Clinical manifestations range from mild symptoms, such as a butterfly-shaped rash and joint pain, to severe complications, including lupus nephritis, a leading cause of morbidity and mortality [41].

Despite advances in treatment, including targeted biologics, many patients fail to achieve therapeutic goals, resulting in end-organ damage and increased mortality risk [42,43,44,45]. For example, a 52-week randomized study demonstrated that weekly subcutaneous belimumab (200 mg), a BAFF inhibitor, significantly improved the SLE Responder Index (SRI-4) response compared to placebo (61.4% vs. 48.4%; *p* = 0.0006) [46]. Another trial evaluating anifrolumab, a type I interferon inhibitor, did not meet its primary endpoint but showed clinically meaningful improvements in patient-reported outcomes [47,48]. Taken together, despite the emergence of targeted biologic therapies, the treatment of SLE remains challenging due to the heterogeneity of the disease and the risk of long-term complications.

In the IM011-021 trial, a phase 2 trial (NCT03252587), 363 adult patients with active SLE were randomized to receive deucravacitinib (3 mg or 6 mg twice daily, or 12 mg once daily) or placebo. At week 32, SRI-4 response rates were significantly higher in the 3 mg (58%) and 6 mg (50%) groups compared to placebo (34%) (odds ratio (OR) 2.8, *p* < 0.001 for 3 mg; OR 1.9, *p* = 0.02 for 6 mg). The 12 mg group showed a nominally higher response rate (45%, *p* = 0.08). Serious AEs were comparable across groups, with no deaths or major complications reported [49].

A systematic review and meta-analysis of cutaneous lupus erythematosus (CLE), a skin manifestation of SLE, found that deucravacitinib was superior to placebo in achieving Cutaneous Lupus Erythematosus Disease Area and Severity Index-50 (CLASI-50), defined as a 50% or greater reduction in the CLASI score (OR: 8.28), and outperformed other treatments, including litifilimab (OR: 2.54) and anifrolumab (OR: 2.25). AEs were comparable among treatments, underscoring deucravacitinib’s promise for CLE patients [50].

Phase 3 trials are ongoing to assess the long-term efficacy and safety of deucravacitinib in active moderate-to-severe SLE: POETYK SLE-1 (NCT05617677) and POETYK SLE-2 (NCT05620407). Additionally, identifying biomarkers for early diagnosis, disease monitoring, and treatment response remains critical to optimizing management strategies and reducing the burden of this debilitating disease.

## 8. Sjögren’s Disease (SjD)

SjD, previously known as Sjögren syndrome, is a chronic autoimmune condition that primarily targets the exocrine glands, especially the lacrimal and salivary glands [51]. Characterized by hallmark symptoms of dry eyes and dry mouth, SjD can also involve systemic complications affecting the lungs, kidneys, and nervous system [52].

The pathogenesis of SjD involves dysregulated immune responses, with key roles played by B cells, T cells, and pro-inflammatory cytokines such as interferons and interleukins (IL-12 and IL-23) [53]. TYK2, a crucial mediator in cytokine signaling for type I interferons and IL-12, is integral to interferon pathways and lymphocyte activation. Genetic variants of TYK2 linked to SjD appear protective, potentially by attenuating signaling from pro-inflammatory cytokine receptors [54].

Current treatment guidelines, as outlined by the European Alliance of Associations for Rheumatology (EULAR), recommend therapies tailored to both local and systemic manifestations. These include topical agents for oral and ocular symptoms, oral muscarinic agonists, hydroxychloroquine, glucocorticoids, synthetic immunosuppressants, and biologics such as rituximab, abatacept, and belimumab [55].

Deucravacitinib, a selective TYK2 inhibitor, offers a novel approach by targeting cytokine pathways involving IL-12, IL-23, and type I interferons. This mechanism may reduce inflammation and glandular damage in SjD. While no clinical trials have yet reported on deucravacitinib in SjD, the ongoing POETYK SjS-1 trial—a phase 3 randomized, double-blind, placebo-controlled study—aims to evaluate its efficacy and safety in adults with active SjD (NCT05946941).

Future research should focus on confirming deucravacitinib’s efficacy in SjD through robust randomized controlled trials. Additionally, combination regimens with existing therapies, such as hydroxychloroquine or biologics, may optimize treatment outcomes. By addressing both glandular and systemic aspects of the disease, deucravacitinib holds promise as a transformative option in SjD management.

## 9. Inflammatory Bowel Disease (IBD)

IBD, including Crohn’s disease and ulcerative colitis, is characterized by dysregulated immune responses involving cytokines such as IL-23, IL-12, and type I interferons. Since the introduction of anti-TNF inhibitors as the first approved treatments for IBD, the roles of the IL-12/Th1 and IL-23/Th17 pathways in IBD pathogenesis have become increasingly recognized. This understanding has led to the approval of biologics such as ustekinumab, guselkumab, and risankizumab for IBD treatment [56,57,58]. Both guselkumab and risankizumab have demonstrated significant efficacy with favorable safety profiles in patients with moderate-to-severe Crohn’s disease [59,60] and ulcerative colitis [61]. Notably, these pivotal trials utilized relatively higher dosing regimens compared to those used for psoriasis.

As deucravacitinib mainly targets IL-12/Th1 and IL-23/Th17 pathways, it is a promising therapeutic agent for IBD. Furthermore, a genome-wide association study identified *TYK2* as a genetic locus associated with susceptibility to Crohn’s disease, indicating that targeting this pathway may help reduce intestinal inflammation in IBD [62].

The LATTICE-UC phase 2 study (NCT03934216) investigated deucravacitinib (6 mg twice daily) in patients with moderately to severely active ulcerative colitis who had inadequate responses to prior therapies. The trial did not meet its primary endpoint of clinical remission at week 12, defined as modified Mayo score with subscores of stool frequency ≤1 with a ≥1-point decrease from baseline, rectal bleeding = 0, and endoscopic subscore ≤1 (modified, excludes friability), with remission rates of 14.8% for deucravacitinib and 16.3% for placebo (*p* = 0.59). While no benefit was observed in biologic-naïve patients, those with prior biologic exposure showed higher remission rates with deucravacitinib (16.1% vs. 0%). Endoscopic response rates, defined as an endoscopic severity subscore of 1 or less, were comparable between groups, and most AEs were mild to moderate in severity [63]. Given the success of higher-dose regimens for biologics of IL-23 inhibitors in IBD, future trials evaluating higher doses of deucravacitinib may reveal improved efficacy.

## 10. Considerations for Deucravacitinib Use in Women

Gender differences in drug response are an important consideration for optimizing the therapeutic potential of deucravacitinib. Hormonal and genetic factors can significantly influence drug metabolism and efficacy. For instance, sex hormones like estrogen can impact the activity of drug-metabolizing enzymes, leading to variations in drug concentration and therapeutic outcomes between genders. Additionally, genetic variations may contribute to different responses among individuals [64,65].

Psoriasis negatively impacts personal, social, and sexual life, with conflicting effects on female fertility, potential pregnancy complications, and limited data on drug safety during pregnancy and lactation [66,67]. Women with moderate-to-severe psoriasis are reported to have more than a 50% reduction in age-adjusted fertility rate compared with the general population [68]. Careful pregnancy monitoring in moderate-to-severe psoriasis is required, given the high risk of pregnancy-related complications, including pregnancy-induced hypertensive disorders, low birth weight for gestational age, and gestational diabetes [67]. Deucravacitinib should generally be avoided during pregnancy due to insufficient data on its safety for pregnant women. Animal studies have not shown evidence of reproductive toxicity, but there are no controlled data in human pregnancy. Furthermore, regarding lactation, there is no information on the presence of deucravacitinib in human milk, its effects on the breastfed infant, or its effects on milk production [69]. When a patient wants to become pregnant or lactate while taking deucravacitinib, she should consult her healthcare provider to discuss potential risks and benefits.

## 11. Limitations

The long-term safety profile of deucravacitinib is not yet fully established, necessitating long-term studies to assess potential risks over extended treatment periods. Cost-effectiveness data for deucravacitinib are currently limited due to its recent approval. However, given its oral administration and the potential reduction in healthcare costs compared to biologics, deucravacitinib could offer a more cost-effective treatment option. Economic evaluations comparing deucravacitinib with existing biologics and JAK inhibitors are needed to determine its value proposition in different healthcare settings. Additionally, there is a lack of direct head-to-head clinical trials comparing deucravacitinib with biologics and JAK inhibitors, which are crucial for drawing definitive conclusions about its relative efficacy and safety. Future research should prioritize these comparative studies to provide clearer insights into deucravacitinib’s positioning within the therapeutic landscape.

## 12. Conclusions

In conclusion, the expanding therapeutic potential of deucravacitinib beyond psoriasis offers a promising approach for treating various immune-mediated diseases. As a selective TYK2 inhibitor, deucravacitinib has demonstrated significant efficacy and a favorable safety profile, solidifying its role as a key treatment option in psoriasis. Emerging evidence suggests potential benefits in conditions such as PsA, PPP, SLE, SjD, and IBD (summarized in Table 1 and Table 2). Although clinical trials for these indications have yielded mixed results, the mechanism of TYK2 inhibition—targeting IL-12, IL-23, and type I interferons—offers a unique advantage over broader immunosuppressive therapies like JAK inhibitors or biologics targeting only one or two cytokines. Additionally, its oral formulation may improve patient adherence, making it a safer and more practical option for long-term management.

Deucravacitinib’s ability to modulate multiple cytokines involved in immune-mediated diseases presents a comprehensive approach to immune-system regulation. This could reduce reliance on combination therapies, which are often associated with higher risks of AEs. Moreover, its selective TYK2 inhibition minimizes off-target effects, distinguishing it from broader immunosuppressive treatments and providing a safer option for patients.

Further research is essential to fully realize the therapeutic potential of deucravacitinib across various conditions. Ongoing and future trials, particularly those evaluating higher doses or specific patient populations, will be critical in defining its role in the treatment landscape. While psoriasis is predominantly driven by the IL-23/Th17/IL-17 axis, other chronic immune-mediated diseases often involve more complex and multifactorial etiologies. Identifying appropriate subgroups or combining deucravacitinib with complementary therapies may optimize outcomes, necessitating carefully designed clinical trials. Additionally, deucravacitinib’s potential to reduce dependence on systemic immunosuppressive treatments could significantly enhance patients’ quality of life by lowering the burden of side effects and improving treatment adherence.

## Figures and Tables

**Table 1:** Summary of efficacy results from the key clinical trials of deucravacitinib.

Trial	Indications	Study Groups	Intervention	Primary Endpoints	Safety (EAIR/100PY or %) (vs. Placebo)
POETYK PSO-1 (phase 3) [14]	Psoriasis	Total (n = 666) Deucravacitinib (n = 332) Apremilast (n = 168) Placebo (n = 166)	Deucravacitinib 6 mg qd, apremilast 30 mg bid or placebo	PASI 75 at week 16: significantly higher with deucravacitinib (58.4%) versus placebo (12.7%) or apremilast (35.1%) (*p* < 0.0001) sPGA 0/1 at week 16 with a ≥ 2-point improvement: significantly higher with deucravacitinib (53.6%) versus placebo (7.2%) or apremilast (32.1%) (*p* < 0.0001)	Any AEs: 211.8 (vs. 202.5) Serious AEs: 7.5 (vs. 19.2) Treatment-related AEs: 33.1 (vs. 45.2)
POETYK PSO-2 (phase 3) [15]	Psoriasis	Total (n = 1020) Deucravacitinib (n = 511) Apremilast (n = 254) Placebo (n = 255)	Deucravacitinib 6 mg qd, apremilast 30 mg bid or placebo	PASI 75 at week 16: significantly higher with deucravacitinib (53.0%) versus placebo (9.4%) or apremilast (39.8%) (*p* = 0.0004) sPGA 0/1 at week 16 with a ≥ 2-point improvement: significantly higher with deucravacitinib (49.5%) versus placebo (8.6%) or apremilast (33.9%) (*p* < 0.0001)	Any AEs: 242.4 (vs. 221.5) Serious AEs: 4.3 (vs. 2.5) Treatment-related AEs: 5.2 (vs. 8.0)
IM011-084 (phase 2) [27]	PsA	Total (n = 203) Deucravacitinib (n = 137) Placebo (n = 66)	Deucravacitinib 6 mg qd, 12 mg qd, or placebo	ACR-20 at week 16: significantly higher with deucravacitinib 6 mg qd (52.9%, *p* = 0.0134) and 12 mg qd (62.7%, *p* = 0.0004) versus placebo (31.8%)	Any AEs: 65.7% (6 mg), 65.7% (12 mg) (vs. 42.4%) Serious AEs: 0% (6/12 mg) (vs. 0%) Treatment-related AEs: 31.4% (6 mg) 25.4% (12 mg) (vs. 9.1%)
IM011-021 (phase 2) [48]	SLE	Total (n = 363) Deucravacitinib (n = 273) Placebo (n = 90)	Deucravacitinib 3 mg bid, 6 mg bid, 12 mg qd, or placebo	SRI-4 response at week 32: significantly higher with deucravacitinib 3 mg bid (58%, *p* < 0.001), 6 mg bid (50%, *p* = 0.02), 12 mg qd (45%, *p* = 0.08) versus placebo (34%)	Any AEs: 93.4% (3 mg bid), 87.1% (6 mg bid), 84.3% (12 mg qd) (vs. 87.8%) Serious AEs: 7.7% (3 mg bid) 8.6% (6 mg bid) 7.9% (12 mg qd) (vs. 12.2%)
LATTICE-UC (phase 2) [63]	IBD (UC)	Total (n = 131) Deucravacitinib (n = 88) Placebo (n = 43)	Deucravacitinib 6 mg bid or placebo	Clinical remission at week 12: not significantly higher with deucravacitinib 14.8% versus 16.3% with placebo (*p* = 0.59)	Any AEs: 70.1% (vs. 47.6%) Serious AEs: 9.2% (vs. 4.8%)

American College of Rheumatology 20 (ACR-20); adverse event (AE); twice a day (bid); exposure-adjusted incidence rate (EAIR); inflammatory bowel disease (IBD); Psoriasis Area and Severity Index (PASI); psoriatic arthritis (PsA); person-years (PY); systemic lupus erythematosus (SLE); SLE Responder Index (SRI-4); once a day (qd); ulcerative colitis (UC).

**Table 2:** Summary of the ongoing key clinical trials of deucravacitinib. Note that primary endpoints were the primary objectives of trials which have not yet been completed or made publicly available.

Trial	Indications	Study Groups	Intervention	Primary Endpoints
POETYK PSO-LTE (phase 3) [16]	Psoriasis	Total (n = 1452)	Deucravacitinib 6 mg qd (open label, extension study)	Incidence of AEs and serious AEs up to 244 weeks (estimated primary completion in 2026-07)
IM011-126 (phase 3) [N/A]	Psoriasis (pediatric)	Total (n = 153; estimated)	Deucravacitinib 3 mg, 6 mg, or placebo	PASI 75 at week 16 in pediatric subjects (age 4–18) with moderate-to-severe plaque psoriasis (estimated primary completion in 2029-01)
POETYK PsA-1 (phase 3) [N/A]	PsA	Total (n = 670)	Deucravacitinib or placebo	ACR 20 at week 16 in participants with active PsA who are naïve to bDMARDs (primary completion 2024-09)
POETYK PsA-2 (phase 3) [N/A]	PsA	Total (n = 729)	Deucravacitinib, apremilast or placebo	ACR 20 at week 16 in participants with active PsA who are naïve to bDMARDs or had previously received TNFα inhibitor: primary completion in 2024-02
POETYK SLE-1 (phase 3) [N/A]	SLE	Total (n = 490; estimated)	Deucravacitinib or placebo	SRI-4 response at week 52: estimated primary completion in 2025-12
POETYK SLE-2 (phase 3) [N/A]	SLE	Total (n = 490; estimated)	Deucravacitinib or placebo	SRI-4 response at week 52: estimated primary completion in 2025-12
POETYK SjS-1 (phase 3) [N/A]	SjD	Total (n = 756; estimated)	Two doses of deucravacitinib or placebo	ESSDAI score at week 52: estimated primary completion in 2026-11

adverse events (AE); biological Disease-Modifying Antirheumatic Drugs (bDMARDs); Sjögren’s Syndrome Disease Activity Index (ESSDAI); not available (N/A); psoriasis area and severity index (PASI); psoriatic arthritis (PsA); Sjögren’s disease (SjD); systemic lupus erythematosus (SLE); SLE Responder Index (SRI-4); once a day (qd).
